# The Comparative Performance of Klypson 500WG and 2GARD-WP Sprayed on Different Wall Surfaces Against *Anopheles gambiae* s.l. in Lower Moshi, Northern Tanzania

**DOI:** 10.3390/tropicalmed10030063

**Published:** 2025-02-27

**Authors:** Maua J. Mohamed, Deokary J. Matiya, Fred D. Chibwana, Winfrida Kidima, Aneth M. Mahande, Eliningaya J. Kweka

**Affiliations:** 1 Department of Zoology and Wildlife Conservation, University of Dar es Salaam (UDSM), Dar es Salaam P.O. Box 35064, Tanzania; greenroseflowers@gmail.com (M.J.M.); deokarijoseph@gmail.com (D.J.M.); fredchibwana@gmail.com (F.D.C.); winikidi@gmail.com (W.K.); 2 Faculty of Science, Dar es Salaam University College of Education (DUCE), Dar es Salaam P.O. Box 2329, Tanzania; 3 Tanzania Plant Health and Pesticide Authority, Pesticides Bioefficacy Section, Arusha P.O. Box 3024, Tanzania; 4 Department of Medical Parasitology and Entomology, School of Medicine, Catholic University of Health Sciences, Mwanza P.O. Box 1464, Tanzania; 5 Bugando Medical Center, Mwanza P.O. Box 1370, Tanzania

**Keywords:** mortality, knockdown, residue activity, bioassays, susceptibility

## Abstract

The emergence of insecticide resistance among malaria vector populations poses a significant threat to existing malaria vector control tools. This phenomenon necessitates an increased pace of developing and deploying new effective compounds in insecticides for vector control. Therefore, this study investigated the comparative performance of newly formulated indoor residual spray compounds, Klypson 500WG (Clothianidin alone) and 2GARD-WP (a mixture of Clothianidin 50% and Deltamethrin 6.25%) against *An. gambiae* in the lower Moshi area of the rural Moshi district, Tanzania. Before the wall cone bioassay tests, the susceptibility of field-collected adult *An. gambiae* s.l. to 0.75% Permethrin, 2% Klypson 500WG, 0.05% Deltamethrin, and 0.25% Pirimiphos-methyl was assessed following WHO procedures. For the cone-bioassay testing, 160 houses were randomly selected and sprayed with Klypson 500WG and 2GARD-WP. For the walls sprayed with Klypson 500WG and 2GARD-WP, the knockdown rate of *Anopheles gambiae* after 60 min of exposure over six months ranged from 70% to 98%, with mortality rates after 24 to 168 h consistently exceeding 90% across all villages and wall types throughout the six months. The susceptibility of wild-collected mosquitoes to Pirimiphos-Methyl, Permethrin, Deltamethrin, and Klypson 500WG was 61, 81, 86, and 93%, respectively. These findings suggest that Klypson 500WG and 2GARD-WP are suitable alternative insecticides that can be incorporated in the vector control toolbox used for malaria control.

## 1. Background

In sub-Saharan African countries, malaria remains a major public health concern [1], primarily affecting children under the age of five and pregnant women [2,3]. Tanzania, excluding Zanzibar Island, is among the ten countries with the highest malaria burden [1], demanding effective strategies for malaria control and elimination [4,5]. The high burden of malaria in sub-Saharan Africa is largely driven by the efficient malaria vectors *Anopheles gambiae*, *Anopheles arabiensis*, and *Anopheles funestus* s.s. [6,7,8].

Several malaria control strategies have been deployed, including Long-Lasting Insecticidal Nets (LLINs) and Indoor Residual Spraying (IRS), which have proven to be highly effective for malaria control [1]. Between 2004 and 2020, the global rollout of LLINs reached 2.3 billion nets, with over 85% distributed in sub-Saharan African countries, including Tanzania [1]. In contrast, population coverage for the IRS was low due to its high cost [9,10]. These insecticide-based strategies have significantly reduced the levels of malaria in mainland Tanzania by more than 30% from 2010 to 2016 [11], while in Zanzibar, the reduction was up to 76% [12].

The major challenge facing vector control strategies is the development of insecticide resistance to malaria vectors. Globally, over 80 malaria-endemic countries have reported resistance of malaria vectors against existing insecticide classes used in vector control. Among those classes, pyrethroids and organochlorines are the most affected [1]. A similar trend of insecticide resistance with different resistance mechanisms in malaria vectors has been reported in Tanzania [6,13,14,15,16,17,18]. The development of insecticide resistance necessitates strategies including alterations, rotations, sequences, mixtures, and mosaics of available insecticides to slow it down or the invention of new insecticides with different or dual insecticides with different modes of action to maintain the effectiveness of intervention attained by these tools.

In response to the wide distribution of insecticide resistance in malaria vectors, the World Health Organization has prequalified new classes for IRS, including Neonicotinoids (NE; Clothianidin and imidacloprid) and butenolides (BU; e.g., flupyradifurone) [4,19,20,21]. Regarding Neonicotinoids, the first Clothianidin formulation was called SumiShield™ 50WG, which showed a novel mode of action by blocking the nicotinic acetylcholine receptors on the postsynaptic membrane of the insect nerve, leading to paralysis [4,22,23], and has prolonged residual efficacy on the sprayed surfaces [22,23,24], making it a good candidate for IRS [23,25,26]. The baseline susceptibility status evaluation of SumiShield™ 50WG against malaria vectors was very high across 16 countries in sub-Saharan Africa [27]. In Tanzania, SumiShield™ 50WG deployment started in 2018 within the Lake Victoria areas, where, in all seven selected sentinel sites, the mosquitoes were fully susceptible up to 2019 [11,28]. However, in 2021, insecticide resistance in malaria vectors was suspected in one of the seven selected sentinel sites, which recorded a mortality below 98% [29].

Moreover, the global plan for insecticide resistance management from WHO has recommended several resistance-delaying strategies for IRS products, including the rotation of insecticides, a combination of interventions, mosaic spraying, and a mixture of insecticide compounds with different modes of action [30,31]. It was further suggested that new or novel insecticide compounds should be mixed with others with varying modes of action to prolong their effectiveness. In response to this requirement, Bayer SAS, developed a new formulation involving a mixture of Clothianidin and Deltamethrin called Fludora^®^ Fusion WG-SB, which the WHO prequalified for IRS in 2018 [5]. It has been deployed in various malaria-endemic areas [22,27,32,33]. Fludora^®^ Fusion WG-SB has shown high bio-efficacy and prolonged residual efficacy against malaria vectors on different types of walls across various malaria-endemic regions [32,33,34,35,36].

Additionally, a new formulation named 2GARD-WP, consisting of WP 500 g/kg Clothianidin and 62.5 g/kg Deltamethrin WP-SB, has been manufactured by Tagros Chemicals India Pvt. Ltd. (Gujarat, India). This new insecticide formulation was prequalified by WHO in 2021 (WHO 2023) and requires further evaluation of its bio-efficacy in the field before its deployment for IRS in respective regions. Therefore, this study investigated the bio-efficacy of Klypson 500WG and 2GARD-WP sprayed on different wall types against *An. gambiae* s.l. in the irrigation schemes area of lower Moshi in northern Tanzania.

Recently, the WHO prequalified two new formulations of Clothianidin 50% WG, Klypson 500WG and 2GARD-WP, consisting of WP 500 g/kg Clothianidin and 62.5 g/kg Deltamethrin WP-SB manufactured by Tagros Chemicals India Pvt. Ltd. However, these new formulations still need further comparative evaluations of their bio-efficacy in the field setting before their deployment in IRS.

## 2. Methods

### 2.1. Study Area

**Figure 1:**
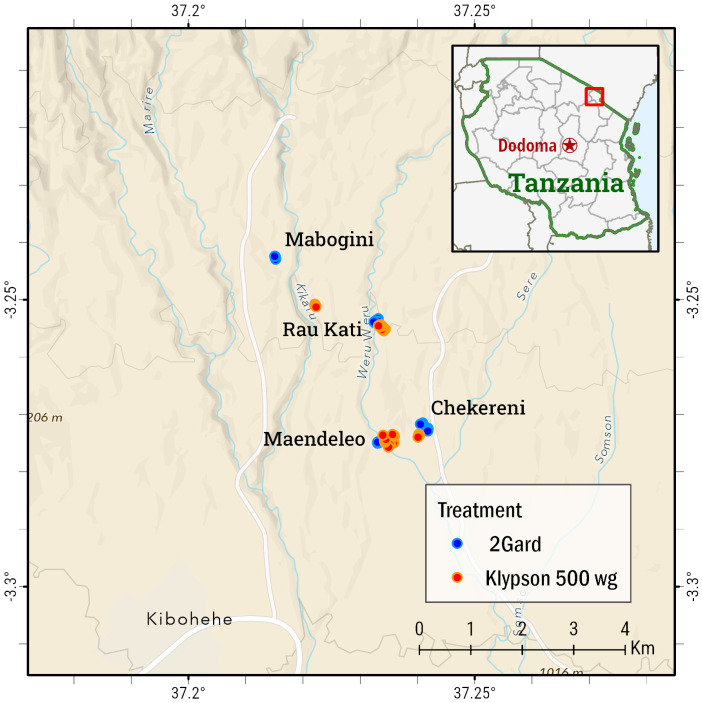
Four villages from Mabogini ward in lower Moshi rice irrigation schemes, rural Moshi district in Kilimanjaro.

This study was conducted in lower Moshi on the slopes of Mount Kilimanjaro (37°20′ E 3°21′ S and 700 m altitude) in north-eastern Tanzania, Mabogini ward. Four villages from Mabogini ward, Maendeleo, Mabogini, Rau-river, and Chekereni, were selected for the study (Figure 1). According to the National Census Report 2022, the population of the Mabogini ward is 57,231 [37]. The main economic activities in this area include rice cultivation, maize farming, and livestock keeping [4,38]. Rice grown under irrigation schemes makes conducive breeding sites for mosquito vectors of malaria, *An. gambiae s.l.* (of which 100% were found to be *An. arabiensis*) [6,39]. Mabogini ward has shown low malaria transmission throughout the year [40]. However, older children and male individuals are the most at risk [41]. The study design was cross-sectional for susceptibility tests and a longitudinal experimental study was carried out for the bio-efficacy tests of treated walls from September 2021 to February 2022.

### 2.2. Mosquito Collection for Susceptibility Test

Mosquitoes were collected indoors from community house structures, including cowsheds, from 6:30 a.m. to 8:00 a.m. They were collected using aspirators and placed into paper cups [42]. The collected mosquitoes were identified to the species level using morphological key developed by Gillies and Coetzee and updated by Coetzee [43,44] and kept in mosquito cages (30 × 30 × 30 cm) and fed 10% sugar solution until the susceptibility test was conducted as per the protocol [30,31,42]. The wild mosquitoes collected that were blood fed were held in cages to be semigravid before they were subjected to susceptibility tests.

### 2.3. Susceptibility Tests

The susceptibility test was conducted following WHO test procedures for susceptibility testing using WHO-impregnated papers with the following concentrations: 0.75% Permethrin, 2% clothianidin, 0.05% Deltamethrin and 0.25% Pirimiphos-methyl [42]. The mosquitoes were exposed for one hour at 10, 15, 20, 30, 40, 50, and 60 min, knocked down, and the mortality rate was recorded. After one hour of exposure, mosquitoes were fed with a 10% sugar solution and evaluated for 24 h post-exposure, and mortality was recorded. The wild population of *An. gambiae* s.l. was collected from cowsheds and human settlements in the lower Moshi rice irrigation scheme area.

### 2.4. Indoor Residual Spraying

IRS was conducted in one hundred and sixty houses which were randomly selected within four villages: Chekereni, Rau River, Maendeleo, and Mabogini. In each village twenty houses were randomly selected for each insecticide (Klypson 500WG and 2GARD-WP). The distance between houses was 10 m or more to avoid any confounding effect, and handheld GPS was used for geo-referencing spraying each house and details. The spraying was conducted following WHO procedures [32]. The monitoring of different wall surfaces sprayed with the insecticides was performed for six months as a standard time to quantify the efficacy of the insecticides being evaluated [32]. Researchers collaborated with district health officers and trained indoor residual spray operators from the Tanzania Plant Health and Pesticides Authority (TPHPA) during the spraying routine. Additional orientation on standard spraying operations and training was given to spray operators three days before they implemented the indoor residual spraying.

Before conducting the indoor residual spraying procedures, the villagers were informed of the study’s specific purpose, importance, and general purposes and signed the consent form.

### 2.5. Preparation of Insecticides for Spraying

The insecticides used for spraying were prepared based on the manufacturer’s instructions. Although the insecticides can be mixed in a separate bucket and tank containing water, the insecticide-wettable granules were poured directly into the water-filled tank to avoid hazards when handling them and mixed in a separate bucket. The prepared insecticides were shaken thoroughly to ensure mixing before spraying started.

All chemicals were provided by Tagros Chemicals India Pvt. Ltd. (Gujarat, India). The 2GARD-WP WP product was contained in sealed water-soluble bags and was applied exclusively on the inner walls of the dwellings at a rate of one 100 g sachet per 10 L sprayer or one 80 g sachet per 8 L sprayer. The target dose rate of the product was 200 mg/m^2^ clothianidin and 25 mg/m^2^ deltamethrin). The Klypson 500WG WG product was intended to be applied exclusively on the inner walls of dwellings at a rate of one 150 g sachet per 250 m^2^. The target dose rate of the product was 300 mg clothianidin/m^2^. The insecticide concentrations were each dissolved in ¾ of 10 litres of water, and were allowed the maximum time to dissolve. Then, the solutions were inserted into 10-litre Hudson X-Pert sprayers ready for the spraying procedures.

### 2.6. Calibration of the Sprayers

The spray team used 10-litre Hudson X-Pert sprayers. The sprayers’ nozzles were checked and calibrated as per WHO guidelines for the Pesticide Evaluation Scheme [30,31]. The flow rate of the constant flow valve used for the six sprayers ranged between 760 and 790 mL/min at a tank pressure of 55 psi. This range was recommended based on the WHO flow rate of 681 to 832 mL/min. Tap water was used, and the pH ranged from 5.5 to 7.0 mg/m^3^, as measured at different points over the 4-day spraying period.

### 2.7. Spraying Procedure

The IRS adhered to the WHO requirements and the insecticides sprayed were 2GARD-WP (500 g/kg clothianidin + 62.5 g/kg Deltamethrin WP-SB) and Klypson 500WG (clothianidin 50%WG) as well as Lambdacyhalothrin [30]. The total number of houses sprayed was one hundred and sixty houses, which were all sprayed within twelve days, and the surfaces of the sprayed walls were burnt bricks, smooth plaster, and rough plaster. Proper disposal of insecticide remains was ensured to avoid polluting the environment and killing non-targeted organisms [45].

### 2.8. Bio-Efficacy Tests of Insecticide-Treated Walls Against Laboratory Colony of An. gambiae

Sixty-four houses were selected for cone bioassays, which were conducted over a period of six months, from September 2021 to February 2022. This was based on the WHO guidelines for monitoring insecticides’ bio-efficacy. Following WHO-standard cone bioassay test procedures (WHO 2013), 15,360 three-day-old female *An. gambiae* mosquitoes obtained from the TPHPA insectary were used for the bio-efficacy tests on the IRS-treated surfaces (House walls). Preparations were conducted in holding rooms with a room temperature of 27 ± 2 °C, and an 80 ± 10% relative humidity was maintained to ensure a conducive environment for mosquito survival [42]. Once all the bioassays were completed, all exposed *An. gambiae* were placed in paper cups and transported to the laboratory to record the overall mortality between 24 and 168 h after exposure [42].

### 2.9. Data Analysis

After cleaning the data in the Excel sheet, an analysis of variance (ANOVA) was performed to compare the mortality rates from the susceptibility tests across the four types of insecticides (Permethrin, Pirimiphos-methyl, Deltamethrin, and Klypson 500WG). Additionally, the bio-efficacy of walls treated with Klypson 500WG and 2GARD-WP was compared between villages, wall types, and months using ANOVA. Mortality rates between the Klypson 500WG and 2GARD-WP treatments were compared using the Tukey Post Hoc test. The KDT_50__ and KDT_95__values were analyzed using a probit analysis. The knockdown time and mortality rate were compared using an independent *t*-test. Data analysis was conducted using SPSS version 27 for Windows.

### 2.10. Ethical Clearence

This study was granted an experimental permit by Tanzania Plant Health and Pesticides Authority with reference No. TPRI/DG/OGC/VOL.XXVI/200.

## 3. Results

### 3.1. Knockdown Time (KDT_50_ and KDT_95_) of An. gambiae s.l During 60 min of Exposure Time in a Susceptibility Test

The KDT_50_ ranged from 38 to 82 min, with the lowest value observed for Klypson 500WG and the highest for Pirimiphos-methyl. Similarly, KDT_95_ ranged between 96 and 140 min, with the lowest value recorded for Klypson 500WG and the highest for Pirimiphos-methyl (Table 1).

### 3.2. The 24 h Mortality Rate of An. gambiae s.l. Post Exposures

**Figure 2:**
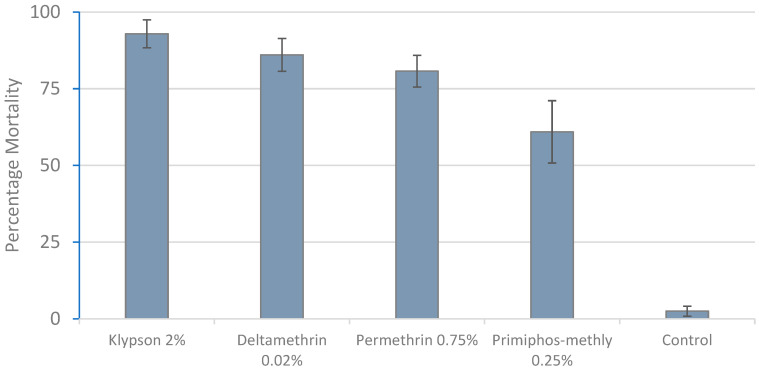
The mortality rate of *An. gambiae* s.l. at 24 hours post-exposure in the susceptibility test from September 2021 to February 2022.

The mortality rates of the four insecticide-treated papers, as shown in Figure 2, ranged from 50 to 93%, with Klypson 2% achieving the highest rate (93%) and Pirimiphos-methyl 0.25% the lowest (61%). Significant variation was observed among the insecticides (*p* < 0.0001, F(4, 110) = 180.36). The Tukey Post hoc test revealed that the mortality rate for Klypson 2% was significantly higher than those for Permethrin 0.75% (*p* = 0.006) and Pirimiphos-methyl (*p* < 0.0001) against the control. Additionally, the mortality rates for Permethrin 0.75% (*p*< 0.01) and Deltamethrin 0.05% (*p* = 0.001) were significantly higher than that for Pirimiphos-methyl 0.25%. However, no significant difference was found between Klypson 2% and Deltamethrin 0.05% (*p* = 0.275) or between Permethrin and Deltamethrin (*p* = 0.580).

### 3.3. The Knockdown Rates for An. gambiae Six Months Post Spray

The knockdown rates after 60 min of exposure are presented in Table 2. For each wall surface, the mortality rates of 2GARD-WP were significantly higher than those of Klypson 500WG across the six months. The knockdown rates of mosquitoes on the walls treated with Klypson 500WG and 2GARD-WP were as follows: On the burned-bricks walls, the highest rates were in September (94 and 98%, respectively) and the lowest in January (70 and 80%, respectively); on the rough plastered walls, the highest rates were in September (95 and 92%, respectively) and the lowest in February (73 and 85%, respectively); and on the smooth plastered walls, the highest rates were in September (87 and 93%, respectively) and the lowest in February (74 and 86%, respectively).

### 3.4. The Overall Mortality Rate of An. gambiae on Insecticide-Treated House Walls in the Villages

**Figure 3:**
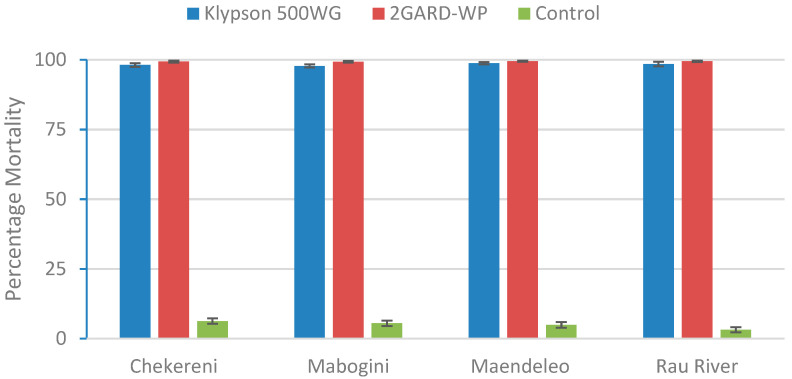
The mortality rate of *An. gambiae* on insecticide-treated house walls in the villages from September 2021 to February 2022.

The overall mortality rate of the mosquitoes exposed to 2GARD-WP was highest across all four villages (Chekereni, Mabogini, Maendeleo, and Rau River), as shown in Figure 3. Furthermore, a comparison of the mortality rates between the two insecticides across these villages showed no significant differences (*p* = 0.120, F(3, 378) = 1.96; ANOVA for Klypson 500WG and *p* = 0.601, F(3, 378) = 0.623; ANOVA for 2GARD-WP).

### 3.5. The Percentage Mortality of An. gambiae Post-Exposure on Insecticide-Treated House Walls

The mosquito mortality rates on various house wall surfaces from 24 to 168 h post-exposure are summarized in Table 3. The mortality rates across all three wall types ranged from 93% to 100%. Both Klypson 500 and 2GARD-WP achieved mortality rates higher than 90% on all wall types. However, 2GARD-WP had significantly higher mortality rates than Klypson 500 on smooth plastered walls at all time points and during the early hours on the rough plastered and burned brick treated walls.

### 3.6. The Effect of Different Wall Types on the Mortality of Exposed An. gambiae

**Figure 4:**
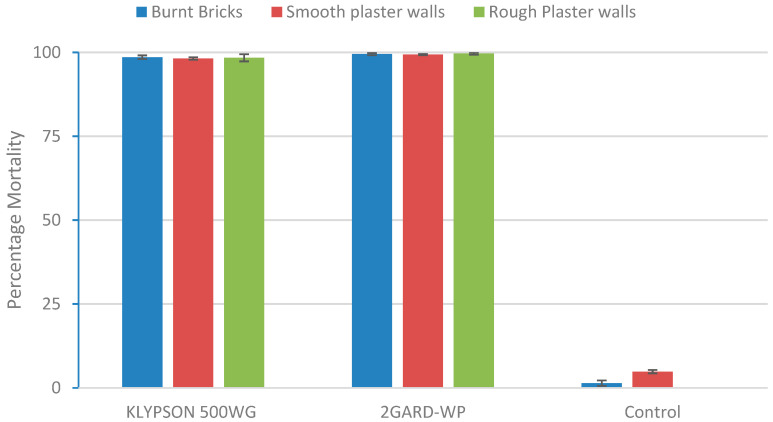
The effect of different wall surfaces on the mortality of exposed *An.gambiae.*

The mortality of mosquitoes on wall surfaces treated with Klypson showed no significant variations (F = 0.471, df = 2 *p* = 0.625), and was similar to that on the 2Gard treated surfaces (F = 0.561, df = 2 *p* = 0.751) (Figure 4). The variation in mortality between the treatment arms and the control was significantly different, with the highest mortality observed for the treatments (F = 13.058, df = 2 *p* < 0.001).

### 3.7. The Percentage Mortality of An. gambiae Post-Exposure over Six Months

The percentage mortality of *An. gambiae* from post-exposure over six months is shown in Table 4. The mortality rates for Klypson 500WG and 2GARD-WP across all post-exposure times during the six months exceeded 90%. In the residual efficacy testing from September 2021 to February 2022, the mortality rates for Klypson 500WG and 2GARD-WP ranged as follows: 88–97% at 24 h, 89–99% at 48 h, 99–100% at 72 h, 99.6–100% at 96 h, 99.6–100% at 120 h, 100% at 144 h, and 100% at 168 h. The mortality rate for 2GARD-WP was significantly higher than that for Klypson 500WG, except in September for those exposed for 24 h (*p* = 0.267) to 168 h (*p* = 0.295).

## 4. Discussion

The findings of this study have shown that both new insecticides are comparatively efficient for vector control with a singular round of IRS for six months. This study evaluated the bio-efficacy of walls treated with Klypson 500WG and 2GARD-WP against *An. gambiae* (Kisumu strain) in rural Moshi, Northern Tanzania. Before testing the treated walls, the susceptibility of wild *An. gambiae* s.l. populations to Klypson 500WG was compared to that for Permethrin, Deltamethrin, and Pirimiphos-Methyl. In the susceptibility testing, following 60 min of exposure, Klypson 500WG had the lowest KDT_50_ (38 min), indicating its high efficacy compared to the other insecticides. This efficacy was similar to that observed in a previous study with SumiShield™ 50WG in the same area [4]. Moreover, after 24 h post-exposure, Klypson 500WG showed a high mortality rate (93%) for *An. Arabiensis*; this mortality rate was slightly higher than the 92% recorded with SumiShield™ 50WG in the same area and other studies conducted elsewhere in Africa [27,46].

On the other hand, our findings indicate that *An. gambiae s.l.* has developed high resistance to other commonly recommended IRS insecticides such as Deltamethrin, Permethrin, and Pirimiphos-methyl. This underscores the suitability of Klypson 500WG, especially when combined with these insecticides, for effectively combating insecticide-resistant malaria vectors in the country. In this study, Klypson 500WG did not achieve full susceptibility (>98%) because Clothianidin as an active ingredient has a delayed mortality effect. This delayed mortality was also observed in studies with SumiShield™ 50WG [22,25,35,47], where 100% mortality was observed between 96 and 168 h post-exposure. Therefore, susceptibility assessments for these insecticides should extend beyond 24 h of post-exposure monitoring.

The cone bio-efficacy of the wall surfaces treated with Klypson 500WG and 2GARD-WP insecticides was analyzed across different villages, wall surfaces, and months, up to six months. The knockdown rate (60 min) of *An. gambiae* exposed to 2GARD-WP was above 80%, while exposure to Klypson 500WG resulted in a knockdown rate below 80%, particularly in the later months of residual efficacy monitoring. The high knockdown rate for 2GARD-WP is attributed to the fast-acting ingredient, Deltamethrin [22]. This highlights the benefit of combining these two insecticides with different modes of action for the IRS.

The assessment of the cone bio-efficacy of Klypson 500WG and 2GARD-WP from 24 to 168 h post-exposure across all four selected study villages showed average mortality rates of more than 97% and 99.5%, respectively. These findings indicate that both formulations, whether with single or mixed active ingredients, are highly effective against malaria vectors in different environments. Although 2GARD-WP was more effective than Klypson 500WG, both exceeded the 80% efficacy cut-off set by the WHO guidelines [48]. These results were similar to those recorded with different formulations of the same insecticides, such as SumiShield™ (Clothianidin) [4,46] and Fludora^®^Fusion WG-SB (Clothianidin + Deltamethrin) [33,36,46].

The 24 to 168 h cone bio-efficacy evaluation based on three types of insecticide-treated walls indicated mortality rates of more than 98% for both Klypson 500WG and 2GARD-WP, demonstrating that these new insecticide formulations are highly effective regardless of the type of house wall. The highest mortality rate of *An. gambiae* in both insecticide formulations was observed on burned-brick and rough plastered walls compared to smooth plastered walls, but this difference was not statistically significant. The higher mortality rates on the burned-bricks and rough plastered walls might be due to their ability to retain insecticides compared to the smooth plastered walls. WP showed superior efficacy to Klypson 500WG across all insecticide-treated house walls. These results highlight the suitability of these insecticide formulations in developing countries, particularly in African settings, where most houses have these types of walls. Consistent with our findings, studies evaluating SumiShield™ 50WG and Fludora^®^Fusion WG-SB in different African countries and India have shown a high efficacy of these insecticide formulations against various malaria vector species across different types of walls [4,23,24,46,49].

The cone bioassay evaluation of residual efficacy over six months demonstrated the high effectiveness of the insecticide formulations Klypson 500WG and 2GARD-WP against *An. gambiae*. The mortality rates were more than 87% for Klypson 500WG and over 92% for 2GARD-WP, indicating robust residual efficacy. Mortality rates increased with longer post-exposure holding times (24, 48, 72, 96, 120, 144, and 168 h), with 2GARD-WP showing significantly higher mortality than Klypson 500WG rates at 24 and 48 h from the third to the sixth month of monitoring. These results are consistent with a study in Benin, where walls treated with SumiShield™ 50WG and Fludora^®^Fusion WG-SB also demonstrated mean mortality rates greater than 80% at post-exposure holding time points of 24 h and beyond, monitored over six months [46].

However, other studies evaluating SumiShield 50 WG have shown varied results. In Tanzania, a study by Kweka and others reported less than 80% mortality at 24 h post-exposure for a susceptible strain of *An. gambiae* over six months [4]. Similarly, studies in Mozambique by Marti-Soler and colleagues [50] and in Ethiopia by Yewhalaw and his team [25] recorded less than 80% mortality at 24 h post-exposure against *An. arabiensis* across the same monitoring period. These discrepancies suggest that the efficacy of insecticide formulations can vary based on geographic location, insecticide formulations, mosquito species, and possibly other environmental factors.

Overall, this study revealed that both Klypson 500WG and 2GARD-WP formulations exhibited a strong residual efficacy against *An. gambiae* on various house walls throughout a six-month monitoring period. These findings underline their potential suitability for integration into the arsenal of indoor residual spraying (IRS) strategies in malaria-endemic regions. The sustained effectiveness of these insecticide formulations highlights their capability to contribute significantly to malaria vector control efforts, emphasizing their importance in combating malaria transmission in the diverse environmental settings commonly found in endemic countries.

## 5. Conclusions

This study evaluated the effectiveness of Klypson 500WG and 2GARD-WP against malaria vectors in rural Moshi, Northern Tanzania, focusing on susceptibility and cone bioassay evaluations. Klypson 500WG demonstrated rapid knockdown with a KDT50 of 38 min, indicating high efficacy compared to the other tested insecticides. Despite not achieving full susceptibility (>98%), it achieved a 93% mortality rate for *An. gambiae* s.l within 24 h post-exposure, surpassing traditional IRS insecticides (Permethrin, Deltamethrin and Pirimiphos-methyl). On the other hand, over six months, Klypson 500WG and 2GARD-WP showed a sustained efficacy in the cone bioassay on *An. gambiae* (Kisumu strain) tests across various wall types. Klypson 500WG consistently achieved mortality rates exceeding 93%, while 2GARD-WP demonstrated over 95%. The higher efficacy of 2GARD-WP is attributed to combining Clothianidin with the fast-acting Deltamethrin component, maintaining mortality rates above 80%. These results underline their high efficacy against *An. gambiae* and suitability for integration into IRS strategies in malaria-endemic regions.

## Figures and Tables

**Table 1:** Knockdown time (KDT) for the mosquitoes exposed to insecticide-treated papers during the susceptibility tests from September 2021 to February 2022.

Insecticide	KDT_50_ (95% CI)	KDT_95_ (95% CI)
Klypson 2%	38.46 (35.96–41.03)	96.34 (91.11–102.37)
Deltamethrin 0.05%	41.92 (39.43–44.50)	99.81 (94.45–105.97)
Permethrin 0.75%	52.49 (49.76–55.44)	110.38 (104.43–117.25)
Pirimiphos-methyl 0.25%	82.08 (77.58–87.10)	139.97 (131.99–149.17)

Key: CI = confidence interval.

**Table 2:** The knockdown rate of *An. gambiae* on treated house walls after 60 min of exposure over six months from September 2021 to February 2022.

Mosquito Knockdown Rate in 60 min After Exposure							
Months	Insecticide	September	October	November	December	January	February
Burned-bricks	Klypson 500WP	94.29	82.95	79.55	76.97	70	77.5
	2GARD-WP	97.50	87.86	82.07	84.52	80.63	82.81
	*p*-value	0.125	0.1	0.514	0.033	0.94	0.073
Plastered-rough	Klypson 500WP	92.26	74.894	75.0	77.143	73.45	72.875
	2GARD-WP	95.00	83.462	84.30	82.58	85.46	85.313
	*p*-value	0.312	0.088	0.002	0.046	0.0001	0.013
Plastered-smooth	Klypson 500WP	86.90	73.49	76.86	75.43	75.25	73.61
	2GARD-WP	92.50	82.73	80.86	83.33	87.14	85.80
	*p*-value	0.053	0.0003	0.304	0.046	0.001	0.0009

**Table 3:** The mean mortality rate of *An. gambiae* post-exposure on insecticides-treated house walls from September 2021 to February 2022.

Wall Type	Insecticide	24 h	48 h	72 h	96 h	120 h	144 h	168 h
Burned bricks	Klypson 500WP	93.2	96.94	99.9	100	100	100	100
	2GARD-WP	100	100	100	100	100	100	100
	*p*-value	<0.0001	<0.0001	0.317	-	-	-	-
Rough plaster	Klypson 500WP	93.8	95	100	100	100	100	100
	2GARD-WP	95	100	100	100	100	100	100
	*p*-value	0.38	<0.0001	-	-	-	-	-
Plastered-smooth plaster	Klypson 500WP	95	97.87	99.2	99.7	99.7	99.8	99.9
	2GARD-WP	99.6	99.3	99.95	100	100	100	100
	*p*-value	<0.001	<0.131	<0.11	0.4	0.208	0.115	0.148

**Table 4:** The mortality rates of *An. Gambiae* from 24 to 168 h post-exposure for six months from September 2021 to February 2022.

Months	Insecticide	24 h	48 h	72 h	96 h	120 h	144 h	168 h
September 2021	Klypson 500WG	92	96.2	99.2	99.6	99.6	100	100
	2GARD-WP	95	98.9	100	100	100	100	100
	*p*-value	0.267	0.185	0.295	0.295	-	-	-
October 2021	Klypson 500WG	92.0	88.5	99.5	100	100	100	98.1
	2GARD-WP	96.3	98.1	99.5	100	100	100	100
	*p*-value	0.009	0.003	0.996	-	-	-	0.616
November 2021	Klypson 500WG	94.4	90.7	99.4	100	100	100	100
	2GARD-WP	97.3	98.6	99.3	100	100	100	100
	*p*-value	0.008	0.019	0.971	-	-	-	-
December 2021	Klypson 500WG	90.6	97.8	99.5	99.8	100	100	100
	2GARD-WP	98.0	99.8	100	100	100	100	100
	*p*-value	<0.001	0.005	0.08	0.319	-	-	-
January 2022	Klypson 500WG	87.7	97.5	99.7	100	100	100	100
	2GARD-WP	93.7	99.7	100	100	100	100	100
	*p*-value	0.008	0.006	0.163	-	-	-	-
February 2022	Klypson 500WG	94.5	98.0	99.5	100	100	100	100
	2GARD-WP	98.3	99.9	100	100	100	100	100
	*p*-value	0.005	0.013	0.081	-	-	-	-

Note: Klypson 500WG (Clothianidin); 2GARD-WP (Clothianidin and Deltamethrin).

## Data Availability

Data are available upon request from corresponding author.
